# Towards Digital Twins of Plasmonic Sensors: Constructing the Complex Numerical Model of a Plasmonic Sensor Based on Hexagonally Arranged Gold Nanoparticles

**DOI:** 10.3390/nano13142044

**Published:** 2023-07-11

**Authors:** Attila Bonyár, Rebeka Kovács

**Affiliations:** 1 Department of Electronics Technology, Faculty of Electrical Engineering and Informatics, Budapest University of Technology and Economics, 1111 Budapest, Hungary; kovacsrebeka@edu.bme.hu; 2 Institute for Solid State Physics and Optics, Wigner Research Centre for Physics of the Hungarian Academy of Sciences, 1525 Budapest, Hungary

**Keywords:** finite element method (FEM), boundary element method, localized surface plasmon resonance (LSPR), plasmonic nanostructure, refractive index sensing

## Abstract

In this work, we aim to design the digital twin of a plasmonic sensor based on hexagonally arranged ellipsoidal gold nanoparticles fixed to a glass substrate. Based on electron microscopy images of three experimentally realized nanoparticle arrangement types, we constructed numerical models in environments based on finite element method (FEM) and boundary element method (BEM), namely COMSOL Multiphysics for FEM and the MNPBEM Matlab Toolbox for BEM. Models with nonperiodic and periodic boundary conditions with different unit cells were constructed to study the plasmonic behavior of both the single ellipsoidal particles and the hexagonal nanoparticle arrangements. The effect of the geometrical parameters, namely the interparticle distance, the nanoparticle diameter and thickness, on the resulting LSPR peak positions and bulk refractive index sensitivities were studied in detail, also taking into account the effect of the SiO_2_ substrate (pillars) under the ellipsoidal particles. We have demonstrated that by optimizing the models, the LSPR peak positions (and sensitivities) can match the experimentally measured values within 1 nm (nm/RIU) precision. The comparison of simulation conditions and the detailed discussion of the effect of the geometrical parameters and used gold dielectric functions on the obtained sensitivities can be very beneficial for the optimization of plasmonic sensor constructions through numerical simulations.

## 1. Introduction

In recent years, label-free detection techniques in biosensing have advanced significantly. Localized surface plasmon resonance (LSPR)-based devices are widely studied due to their small size and real-time detection of biomolecular interactions [1]. One of the greatest advantages of LSPR-based sensors is their excellent sensitivity to changes in the refractive index of the surrounding medium [2]. Surface plasmon resonance (SPR)-based biosensors have been used in gas and water sensing [3,4], nucleic acid detection [5] and biosensing [6]. Localized surface plasmon polaritons are the collective free electron oscillations at the gold nanoparticle surface excited by an external electric field (where the wavelength is longer than the characteristic size of the nanoparticle). This effect can result in the enhancement of scattering and absorption of the incident electromagnetic waves, and due to its sensitivity to the surrounding medium’s refractive index, it can be successfully utilized for sensing purposes [7]. 

In the past years, a novel surface nanocomposite sensor was developed in the framework of a collaboration that consists of hexagonally ordered, ellipsoidal gold nanoparticles bound to an epoxy surface on a large (cm^2^) surface area [8]. Although this sensor was successfully utilized as a working nucleotide sensor for DNA detection [8], preliminary calculations performed by boundary element method (BEM) simulations showed that by optimizing the geometrical properties of the nanoparticle arrangement, its sensitivity could be increased significantly [9]. In this work, we aim to construct the digital twin of these nanoparticle arrangements. For this, here, we primarily rely on the FEM approach, which enables the utilization of periodic boundary conditions, which was not previously possible in our investigations with the BEM method (using the MNPBEM Matlab Toolbox [10]). Our specific aim is to model and reconstruct three experimentally realized arrangements and use the constructed digital twin to study their plasmonic behavior through numerical simulations. These insights could help in the optimization of their fabrication technologies in order to maximize their performance for various sensing applications. Besides sensors, the developed nanocomposites are also aimed to be used as targets in the Nanoplasmonic Laser Fusion Research Laboratory (NAPLIFE) project [11,12]. 

## 2. Methods

### 2.1. The Investigated Nanoparticle Arrangements

**Figure 1:**
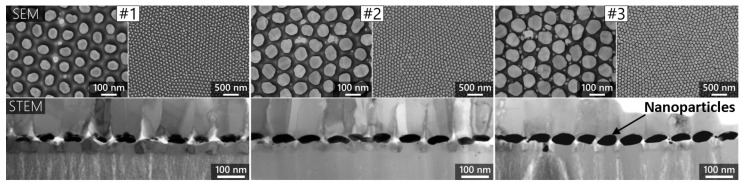
The three different nanoparticle arrangements (type #1, #2, and #3). The top images present SEM images made while the nanoparticles were over the aluminum template after synthesis. The bottom STEM (BF) images were obtained on the final sensor elements, with the NPs already on top of the SiO_2_ nanopillars/substrate. Image reproduced from [13].

In this work, three different hexagonal arrangements containing ellipsoidal nanoparticles are investigated. All three arrangements can be realized experimentally by using the combination of porous anodic alumina (PAA) preparation, thin film deposition and subsequent solid-state dewetting (SSD) as discussed in our previous publications [8,13]. In the current technology, the nanoparticles are transferred by depositing a SiO_2_ layer onto the particles in a PECVD chamber, then gluing the (Al/AuNPs/SiO_2_) structure to a glass slide with a two-compound epoxy glue (Loctite 3430). SEM and STEM images from the three particle arrangements and the resulting nano-mushroom structures are presented in Figure 1. The evaluation of the AuNP size was performed by image processing software (Adobe Photoshop, Gwyddion) as discussed in Supplementary Materials of ref. [13]. The nominal geometrical parameters of the investigated arrangements are given in Table 1. Here, the interparticle gap (*D*) was calculated as the difference between nanopore diameter (*D*_p_ = 111 nm for all three arrangements) and nanoparticle diameter (*D*_0_). In the numerical models the ellipsoids were defined by two axes and their ratio *c*, where *c* = diameter (*D*_0_)/thickness (*t*), with nm precision. The dimensions of the nanoparticles are shown in Table 1. For more information regarding the fabrication technology, see papers [8,13]. 

### 2.2. Simulation Details

**Figure 2:**
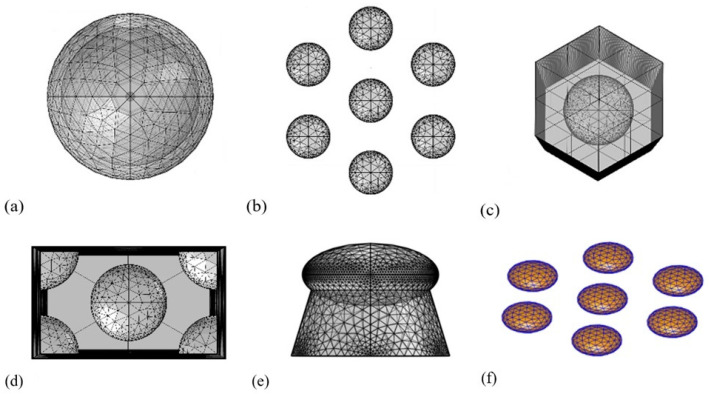
Illustration of the different investigated models and their meshing, in accordance with the numbering of Table 2. (**a**) Nonperiodic single particle inside a spherical PML (no. 1). (**b**) Nonperiodic hexagonal particle arrangement (no. 2, spherical PML not visible). (**c**) Periodic boundary condition, hexagonal unit cell (no. 4), (**d**) Periodic boundary condition, square unit cell (no. 3). (**e**) Periodic model with the added substrate (no. 7), and (**f**) hexagonal BEM model (no. 6).

The three nanoparticle arrangements were investigated by different models created in FEM- and BEM-based solvers. Table 2 summarizes the parameters and conditions used to set up the different models, while Figure 2 illustrates the structures and the meshing. 

The FEM-based 3D modeling and numerical simulations were performed using the Wave Optics module of COMSOL Multiphysics 3.5 software. The solver solves Maxwell’s equations on the E-field within the computational domain, as in Equation (1), where *k*_0_ is the free space wave number, and *σ* and *μ_r_* are the conductivity and relative permeability of the medium, *ω* is the frequency of the incident light, *ε*_0_ is the permittivity of free space and *ε_r_* is the relative permittivity, respectively [14].

(1) $$\nabla *\mu_{r}^{-1}\left(\nabla *E\right)-k_{0}^{2}\left(\varepsilon_{r}-\frac{j\sigma }{\omega \varepsilon_{0}}\right)E=0.$$

The dielectric functions used to model the gold nanoparticles were based on the experimental results of McPeak et al. [15]. 

For BEM-based simulations, the MNPBEM Matlab Toolbox was used, which was specifically designed to study the plasmonic behavior of metallic nanomaterials [10]. The BEM solver was configured as discussed in our previous works [9]. In short, the nano-ellipsoids were created by scaling nanospheres created by the ‘trisphere’ function with 144 vertices (boundary elements). The retarded solver was used which solves the full Maxwell equations on these elements, also considering McPeak’s dataset for modeling gold. 

With both solvers, the solutions of Maxwell’s equations were further processed to obtain the extinction cross-sections. The optical cross-sections of the sub-wavelength particles are evaluated as in Equation (2), where *P_inc_* is the incident irradiance, defined as energy flux of the incident wave [W/m^2^], *W_abs_* is energy rate absorbed by the particle [W], *W*_sca_ is scattered energy rate [W] [14].

(2) $$\sigma_{abs}=\frac{W_{abs}}{P_{inc}},\;\;\;\;\sigma_{sca}=\frac{W_{sca}}{P_{inc}}.\;\;\;\;$$

The total extinction cross-section is the amount of energy removed from the incident field due to scattering and absorption, as in Equation (3).

(3) $$\sigma_{ext}=\sigma_{abs}+\sigma_{sca\;},\;\;\;\;\;\;\left[m^{2}\right].\;\;\;\;\;\;$$

With both solvers and for all nanoparticle arrangements, LSPR sensing performance was evaluated based on nanoparticle response to the refractive index changes in the surrounding medium. The bulk refractive index sensitivity (*RIS*) is thus defined as in Equation (4), where $\Delta \lambda_{p}$ is the peak shift of the extinction spectrum upon changing the refractive index of the dielectric medium. For this, the dielectric environments surrounding the nanoparticles were modeled with a constant refractive index (e.g., air *n*_1_ = 1 and water *n*_2_ = 1.33, thus Δn=0.33).

(4) $$RIS=\frac{\Delta \lambda_{p}}{\Delta n\;}.$$

To analyze the correlation between the nanoparticle arrangement, their coupling efficiency, and the refractive index sensitivity, the performance of single particles (Model no. 1 and 5 in Table 2) was compared with the performance of hexagonal particle arrangements (Model no. 2 and 6) with both solvers. As it is not possible to use a periodic boundary condition in the BEM solver, these FEM simulations (Model no. 1 and 2) serve as a comparison between the two solver approaches. In these hexagonally arranged nanoparticle simulations, the nanoparticles were placed 111 nm from each other (center to center) with geometrical parameters defined by Table 1. Additionally, in the FEM solver, perfectly matched layers (PMLs) were constructed in the models in order to absorb the incident wave and the reflections. For Models no. 1 and 2, these PMLs were spheres with a diameter of 1200 nm, as illustrated in Figure 2a for a single particle. 

To utilize the advantage of FEM, two models with periodic boundary conditions were also constructed (Models no. 3 and 4), one with a hexagonal (Figure 2c) and one with a rectangular unit cell (Figure 2d). Both the design and the computational method of the two periodic models were based on the work of Borah et al. [16]. In these two cases, the PMLs were above and below the plane of the particle arrangements at a distance between 1000–2000 nm from the plane of the particles (depending on the size of the particles).

For BEM simulations and FEM simulations with nonperiodic boundary conditions, the nanoparticles were excited by a plane wave with linear polarization, with propagation direction perpendicular to the plane of the particle arrangement and linear polarization angle parallel to the main coupling direction of the particles. We used circular polarization for FEM simulations with periodic boundary conditions, which reproduced the linearly polarized excitation with a 0.1 nm precision.

To consider the effect of the SiO_2_ substrate, a model with SiO_2_ pillars under the nanoparticles was also constructed, as in Figure 2e. The geometrical parameters of this pillar were estimated based on the STEM images of Figure 1, and thus the top and bottom diameter and height of the cone were scaled according to the diameter and thickness of the nanoparticles. The refractive index of the pillar was selected to be 1.45.

## 3. Result and Discussion

In the first subsection, we compare the different simulation models, while in the second subsection we focus on the comparison with experimental results.

### 3.1. Comparison of the Investigated Models

The resulting LSPR peak positions and the bulk refractive index sensitivity are collected in Table 3 for the seven model types and three nanoparticle arrangements. The similarities and differences in the results for the different models are discussed below.

#### 3.1.1. BEM vs. FEM Solver

**Figure 3:**
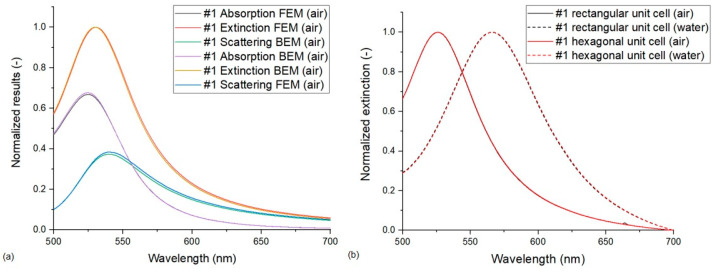
Simulation results on type #1 nanoparticle arrangements. The compared conditions are the following: (**a**) extinction, absorption and scattering cross-sections calculated with FEM and BEM solvers (Model no. 2 vs. 6). (**b**) Normalized extinction cross-sections calculated on rectangular and hexagonal unit cells with periodic FEM models (Model no. 3 vs. 4).

The two approaches were compared for both single particles and hexagonal seven-particle arrangements. It was found that the LSPR peak positions and the resulting sensitivity values match each other within 1 nm precision for the single-particle simulations and for the hexagonal arrangements, except for type #3, which resulted in a ~1 nm difference in the peak positions. Resulting extinction, absorption and scattering cross-sections for the type #1 hexagonal particle arrangement are also compared in Figure 3a. We can conclude that the results gained by the two solvers are well comparable when the same dataset is used for modeling the dielectric function of gold, in our case, McPeak’s. However, the FEM solver has some direct advantages such as the periodic boundary condition or the flexibility in designing the substrate geometry. 

#### 3.1.2. Rectangular vs. Hexagonal Unit Cells in Periodic FEM Models

A similar good agreement was found between the two investigated FEM models that used periodic boundary conditions (Model no. 3 vs. 4). For type #1 arrangement, the differences in the peak positions were less than 1 nm for the results obtained with the rectangular and hexagonal unit cells, as illustrated in Figure 3b. For type #2 and #3 arrangements, there were peak position differences in the 1 nm range that only translated into a 3 nm/RIU difference in sensitivity for type #3 arrangement. According to Borah et al. [16], the good correspondence between the results obtained with the two different unit cells confirms that the models are configured well, and the periodic boundary conditions are set properly. Since the number of elements in the model with a hexagonal unit cell is significantly smaller, this was used for all further calculations with periodic boundary conditions.

#### 3.1.3. Periodic vs. Nonperiodic FEM Models

**Figure 4:**
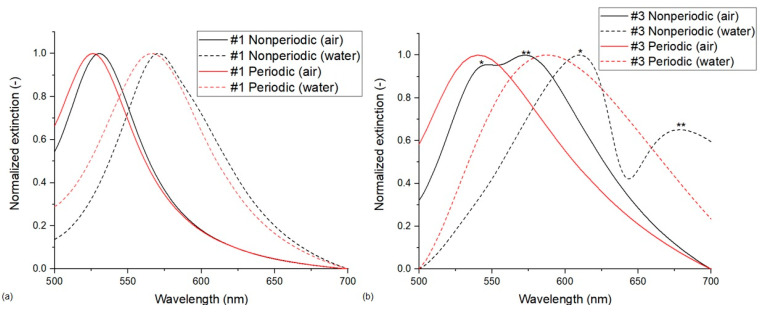
Normalized extinction cross-sections calculated with periodic (hexagonal unit cell) and nonperiodic FEM simulations (Model no. 2 vs. 4). (**a**) Type #1 nanoparticle arrangements. (**b**) Type #3 nanoparticle arrangements. In (**b**), the * peak marks the ‘outer’ resonance mode, while ** marks the ‘inner’ mode, as illustrated in Figure 5.

**Figure 5:**
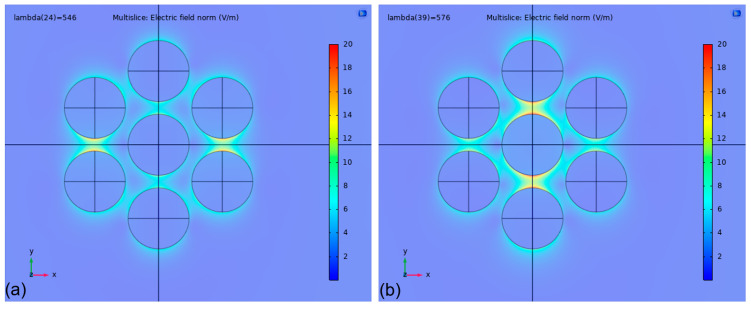
Normalized electric field strength maps, calculated with the nonperiodic, hexagonal FEM model (Model no. 2) on type #3 nanoparticle arrangement at *n* = 1 (in air). The linear polarization vector is parallel to the y-axis. (**a**) Field strength at *λ*_p_ = 546 nm, corresponding to the ‘outer mode’. (**b**) Field strength at *λ*_p_ = 576 nm, corresponding to the ‘outer mode’.

Significant differences were observed between models running with periodic and nonperiodic boundary conditions. As we mentioned, there were good agreements between the two FEM periodic models with different unit cells and also between the FEM and BEM nonperiodic models. Therefore, in this section, we can generally compare Models no. 3 and 4 against Models no. 2 and 6 (see Table 2). The difference between the two boundary conditions becomes more pronounced as the *D*/*D*_0_ value of the arrangement decreases, or, in other words, as the coupling between the nanoparticles increases with decreasing interparticle distance. The reason for the difference is the retardation of the extinction spectrum which is illustrated in Figure 4 for type #1 (a) and type #3 (b) arrangements, respectively. The appearance and separation of two resonance modes are only present in the nonperiodic simulations. In Figure 5, electric field strength maps demonstrate the two resonance modes corresponding to the two peaks observable in Figure 4b for type #3 arrangement, calculated in air. As can be seen in Figure 5, the peak at *λ*_p_ = 546 nm corresponds with an ‘outer’ resonance mode, while the peak at *λ*_p_ = 576 nm represents an ‘inner’ mode with respect to the center of the arrangement. This retardation of the spectrum is barely noticeable for type #1 arrangement, except for a slight shoulder in the spectrum calculated in water (green curve in Figure 4a), but it becomes more pronounced as the *D*/*D*_0_ decreases. For type #2 and type #3 arrangements, the two modes are easily distinguishable in the spectra. In this work, peak positions and sensitivity values in Table 2 were always calculated for the peak at a lower wavelength (corresponding with the ‘outer’ mode). The clear separation of the two modes causes an increased sensitivity for arrangement type #3, even for this mode (195 nm/RIU). In the event of calculating with the ‘inner’ resonance mode at longer wavelengths (marked with ** in Figure 4b), the sensitivity would increase to 267 nm/RIU and 330 nm/RIU for type #2 and #3 arrangements, respectively.

The retardation of the spectra is visibly suppressed in the models with periodic boundary conditions. The width of the resulting spectra is larger, but no mode separation can be observed, even at lower *D*/*D*_0_ values. As discussed in Section 3.2 in more detail, the lack of peak separation and the resulting sensitivity values approximate the experimental spectra and sensitivity values far better. Thus, for all further calculations, the periodic FEM models (with hexagonal unit cells) are used, as we consider them superior compared to the other approaches.

#### 3.1.4. Single Particles vs. Hexagonal Arrangements

**Figure 6:**
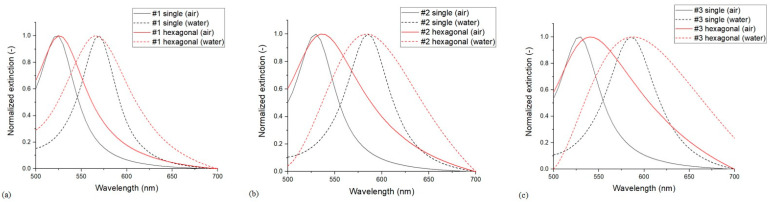
Normalized extinction spectra of the single particle and the periodic hexagonal FEM models (Model no. 1 vs. 4) in air and water for (**a**) type #1 (**b**) type #2 and (**c**) type #3 nanoparticle arrangements.

By comparing the results obtained on single-particle models (Model no. 1 or 5) with hexagonal models (Models 2, 3, 4 and 6), we can see that the sensitivity of the hexagonal arrangements decreases significantly, regardless of the boundary conditions (see Table 3). As illustrated in Figure 6 for the three different arrangements, with the hexagonal unit cell, the full width at half maximum (FWHM) of the spectra increases significantly; in addition, there is a redshift in the spectra calculated in air, which accounts for the loss in sensitivity. In a previous work, where coupled nanoellipsoid dimers were investigated with a BEM approach, no such decrease in sensitivity was observed at this *D*/*D*_0_ range compared to single particles [9]. This indicates that the coupled mode between the hexagonally arranged particles differs from the dimers, and here, smaller *D*/*D*_0_ values are required to utilize the positive effect of near-field coupling between the particles and increase the sensitivity of the arrangements. This is elaborated further in Section 3.2.

#### 3.1.5. The Effect of Substrate

**Figure 7:**
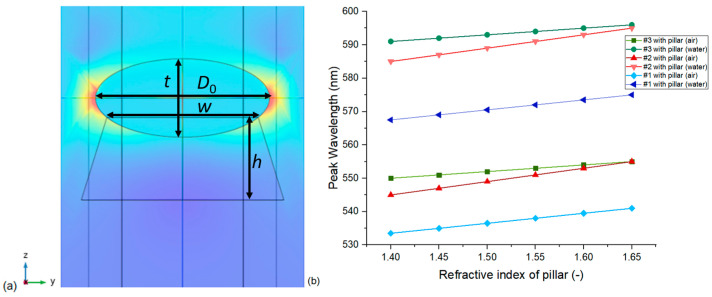
(**a**) Normalized electric field strength map, calculated with the periodic, hexagonal FEM model (Model no. 4) on a type #3 nanoparticle arrangement at *n* = 1 (in air), with an SiO_2_ pillar (*n*_s_ = 1.45). The labels mark the main geometrical parameters. (**b**) The effect of the pillars’ refractive index on the LSPR peak positions in air and water for the different nanoparticle arrangements.

The effect of the SiO_2_ pillars under nanoparticles was only studied for the periodic FEM model with a hexagonal unit cell. The inclusion of the pillars resulted in a ~20 nm/RIU drop in the obtained sensitivity values for the three nanoparticle arrangements. This is as expected since the pillars fill a significant area under the nanoparticles with a fixed and higher refractive index medium, thus red-shifting the resonance peaks and causing decreased responsivity. In all further investigations in Section 3.2, this periodic model with the substrates is used and compared with the experimentally obtained spectral data. It is important to mention that the pillar geometry was designed based on the STEM images of Figure 1, and they are scaled with the actual geometry of the nanoellipsoids. Thus, as the height of the pillar (*h*) is larger than the effective penetration depth of the plasmon field in this direction (see Figure 7a), small variations in the actual geometry of the pillars do not have a significant effect on the resulting spectra.

On the other hand, the refractive index of the pillar material has to be considered critically. For the results in Table 2, the RI of the deposited SiO_2_ pillars was selected to be 1.45, but as can be seen in Figure 7b, this RI naturally affects the peak positions. It is important to note that changing the RI of the pillars does not influence the calculated sensitivity, only the peak positions (these simulations were performed with 0.5 nm rounding precision). For all further simulations, *h* = 1.3 × *t* and *w* = 0.8 × *D*_0_ are used, but the effect of particle embedment, namely the *w*/*D*_0_ ratio, is investigated in Section 3.2.3.

### 3.2. Comparison of the Experimental and Simulation Results

In order to fine-tune our FEM model, the experimental response of the three different nanoparticle arrangements was measured with a spectrophotometer by changing the refractive index inside the channel between *n* = 1 (air) and *n* = 1.33 (water). All samples were cleaned by oxygen plasma treatments, as discussed in [8]. Table 4 collects the resulting average LSPR peak positions in air and average sensitivities.

By comparing these results with those of Model 7 in Table 3 (FEM model with periodic boundary condition and hexagonal unit cell, including the SiO_2_ pillars), we can note two important differences. Both the measured peak positions in air and the sensitivities are higher compared to the calculations. Concerning the sensitivities, the difference is negligible for type #3 arrangements (1 nm/RIU), small for type #2 (7 nm/RIU), and significant (21 nm/RIU) for type #1 particles. The difference between the peak position in air is always significant (between 28 and 44 nm). This discrepancy likely arises from the irregular shape and geometrical variation of the nanoparticles (see Figure 1 and Table 1). The particle diameter (*D*_0_), the interparticle gap (*D*), the diameter/thickness ratio (*c*), and the refractive index of the SiO_2_ pillars (*n*_s_) all have an effect on the resulting peak positions and sensitivities. The experimental variation of these geometrical parameters is also well-reflected in the deviation of the obtained peak position and sensitivity values in Table 4. The effects of these parameters on the resulting plasmonic properties are studied individually in the next subchapters. 

#### 3.2.1. The Effect of *D*/*D*_0_

**Figure 8:**
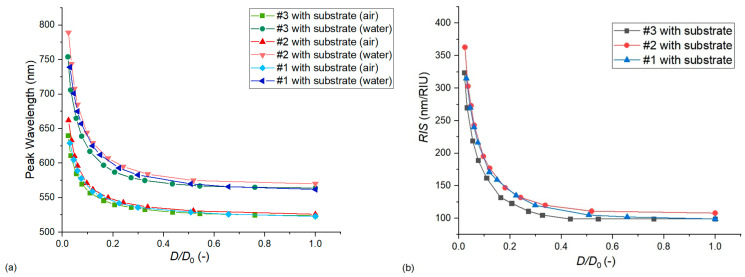
The effect of the dimensionless interparticle gap/particle diameter ratio (*D*/*D*_0_) on the LSPR peak wavelength (**a**) and bulk refractive index sensitivity (**b**). Calculations were performed with Model no. 7 (see Table 2) by changing the interparticle distance and keeping the nanoparticle geometry (diameter and thickness) fixed.

**Figure 9:**
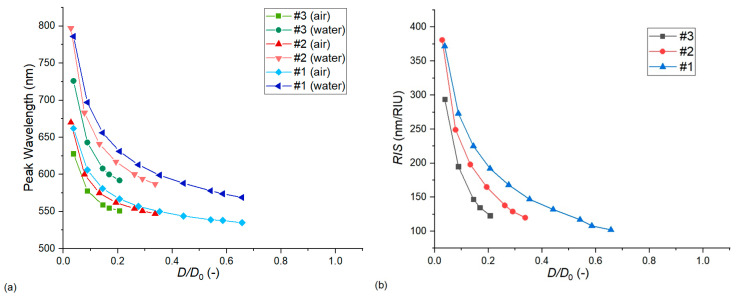
The effect of the dimensionless interparticle gap/particle diameter ratio (*D*/*D*_0_) on the LSPR peak wavelength (**a**) and bulk refractive index sensitivity (**b**). Calculations were performed with Model no. 7 (see Table 2) by keeping the pore diameter fixed while increasing the particle diameter (and thus also the diameter/thickness ratio).

As discussed in previous works, the interparticle gap/particle diameter ratio (*D*/*D*_0_) is an important dimensionless quantity that characterizes the effect of near-field coupling and sensitivity increase between closely packed nanoparticle assemblies [9,17,18]. In our case, we can study its effect via two approaches. In Figure 8, *D*/*D*_0_ is modulated by changing only the interparticle distance (*D*) and keeping the geometry of the nanoparticles constant. This means a fixed *D*_0_ and also a fixed diameter/thickness ratio (*c*). As the interparticle distance is defined as the difference between the pore diameter and the particle diameter (*D* = *D*_p_ − *D*_0_, as defined in Section 2.1), experimentally, this would mean the modulation of the pore diameter, which cannot be realized due to the constraints of the template [8]. Thus, in Figure 9, the effect of *D*/*D*_0_ modulation is also presented by keeping the pore diameter fixed at *D*_p_ = 111 nm and changing only the particle diameter (*D*_0_). As the particle thickness is also held constant, this changes the diameter/thickness ratio (*c*). All simulations were performed with Model no. 7.

Both Figure 8 and Figure 9 confirm that the hexagonal arrangements behave according to the well-established plasmon ruler law, by decreasing *D*/*D*_0_, the LSPR peak position and sensitivity increases exponentially. However, this increase was much steeper in the case of Figure 9, where the particle diameter was increased, as this also increased the anisotropy of the particle and the diameter/thickness ratio (*c*), resulting in an additional sensitivity increase. As for the difference between the experimental and simulated sensitivities, we can see in Figure 9 that for type #2 arrangements, only a small increase in the particle diameter can increase the sensitivity to match the experimental results of Table 4 (namely 2 nm, which corresponds to decreasing *D*/*D*_0_ to 0.31 from 0.33, by keeping the particle thickness fixed). This is well within the uncertainty of particle diameter measurement and its variation (see Figure 1 and Table 1). For type #1 arrangement, the discrepancy of 123 nm/RIU and 102 nm/RIU would require increasing the particle diameter by around 7 nm to find a match between the simulation and the experiment. Also, although decreasing *D*/*D*_0_ increases the LSPR peak wavelength position, in this range, this cannot account for the huge differences between the experimental and simulated peak positions.

We can also see that due to the exponential relationship between the sensitivity and *D*/*D*_0_, if we would take into consideration the distribution of particle dimensions (as in Table 1), it would lead to increased *RIS* values, as in a distribution, the larger particles would contribute more compared to particles with homogenous (nominal) geometry.

#### 3.2.2. The Effect of the Diameter/Thickness Ratio (*c*)

**Figure 10:**
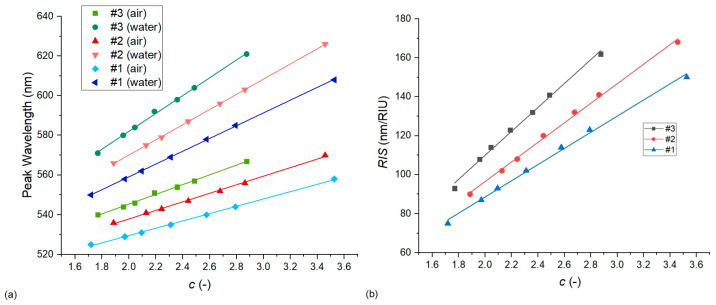
The effect of the nanoparticle diameter/thickness ratio (*c*) on the LSPR peak wavelength (**a**) and bulk refractive index sensitivity (**b**). Calculations were performed with Model no. 7 (see Table 2) by changing only the nanoparticle thickness (in the −10 nm–10 nm range) and keeping the particle diameter constant.

The effect of this parameter was also studied along two approaches. In the first, illustrated in Figure 10, only the thickness of the nanoellipsoids was changed (in the −10 nm–10 nm range) while keeping the particle diameter (*D*_0_) and thus the *D*/*D*_0_ ratio fixed. Again, we can see that the *RIS* values are quite sensitive to the variations in the thickness and *c* ratio in the range of our experimental uncertainty (around ±4–6 nm in the thickness). For example, a decrease of 2 nm in the thickness of particles in type #2 arrangements (corresponding to an increase in the *c* ratio from 2.44 to 2.59) could increase the calculated sensitivity to match the experimental results. For type #1 arrangements, this would again require a larger compensation, namely a decrease of ~5 nm in thickness.

**Figure 11:**
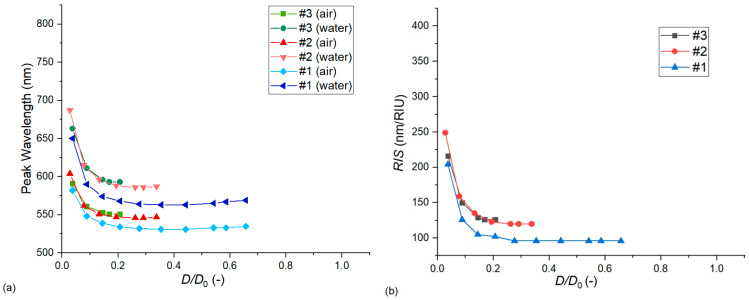
The effect of the nanoparticle diameter/thickness ratio (*c*) on the LSPR peak wavelength (**a**) and bulk refractive index sensitivity (**b**). Calculations were performed with Model no. 7 (see Table 2) by increasing both the particle thickness and diameter simultaneously with the same increments in the 0–40 nm range for type #1, 0–25 nm range for type #2, and 0–15 nm range for type #3 arrangements.

In the second approach illustrated in Figure 11, the particle diameter and thickness were increased simultaneously with the same amount, gradually, in the 0–40 nm range for type #1, 0–25 nm range for type #2 and 0–15 nm range for type #3 arrangements. This approach also has experimental relevance as the size of the nanoellipsoids could be increased after their synthesis in a controlled manner, e.g., by using wet chemical methods to reduce gold on the nanoparticles from HAuCl_4_ precursors [19]. This method, namely the homogenous deposition of gold on the particles, increased the diameter and the thickness of the particles simultaneously. As the growth of the nanoparticles also decreased the *D*/*D*_0_ ratio, the results were plotted in its function in Figure 11. We can see that the effect of decreasing *D*/*D*_0_ was compensated by the decreasing *c* value, and thus our exponential characteristics were less steep than those of Figure 9, where the particle diameter was increased by keeping the thickness constant. 

**Figure 12:**
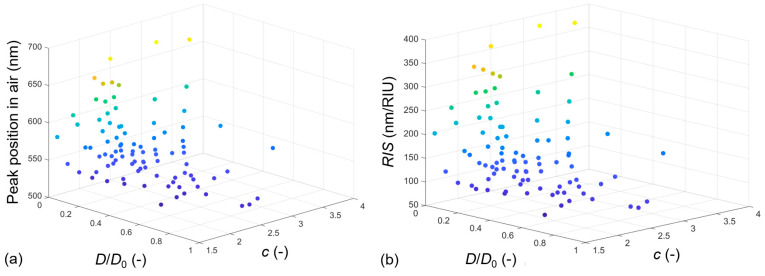
LSPR peak wavelength (**a**) and bulk refractive index sensitivity (**b**) in the function of the dimensionless interparticle gap/particle diameter ratio (*D*/*D*_0_) and the diameter/thickness ratio (*c*). Results are cumulated for all investigated scenarios in this paper with Model no. 7, namely a complete number of 91 parameter combinations. The color information corresponds with the z-axis values, namely with the peak position in air (**a**) and *RIS* (**b**).

We saw that both the interparticle gap/particle diameter ratio (*D*/*D*_0_) and the diameter/thickness ratio (*c*) have a substantial effect on both LSPR peak positions and resulting sensitivities. Their cumulative effect is presented in the heat maps of Figure 12 for peak positions in air and *RIS* values. 

#### 3.2.3. The Effect of Substrate Geometry and Level of Particle Embedding

**Figure 13:**
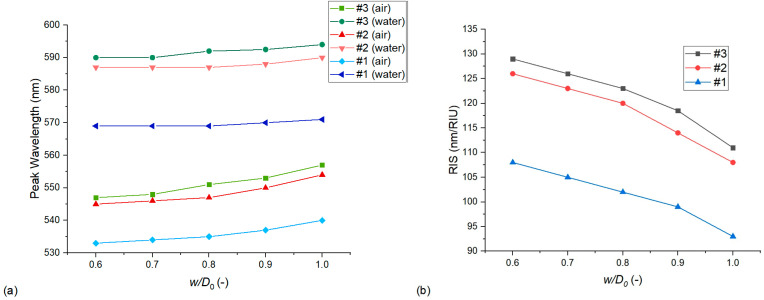
The effect of the substrate’s geometry on the LSPR peak wavelength (**a**) and bulk refractive index sensitivity (**b**). Calculations were performed with Model no. 7 (see Table 2) by changing the *w*/*D*_0_ ratio, where *w* is the upper diameter of the conical SiO_2_ pillar and *D*_0_ is the diameter of the ellipsoidal nanoparticles (see Figure 7a).

As mentioned in Section 3.1.5, the SiO_2_ pillars under nanoparticles were designed based on the STEM images of Figure 1. The height of the pillar (*h*) was selected to be larger than the thickness of the particles (*t*, and *h* = 1.3 × *t*) and thus its variation did not affect the simulation results (data not shown). However, the level of particle embedding, namely the surface area of the nanoparticle, which is covered by the substrate, had a significant effect on both LSPR peak position and sensitivity. This embedment was characterized by the *w*/*D*_0_ ratio, as illustrated in Figure 7a, where *w* is the upper diameter of the conical pillar, and *D*_0_ is the diameter of the particle. Figuratively, at *w* = *D*_0_, exactly 50% of the nanoparticle’s surface area (the lower half of the particle) is embedded into the SiO_2_ pillar. Considering this *w*/*D*_0_ = 1 as an upper limit, Figure 13a,b present its effect on the LSPR peak wavelength and sensitivity, respectively. As expected, pushing the particles deeper into the substrate the peak wavelength increases (as the SiO_2_ pillar has a higher RI compared to the surrounding medium), while the sensitivity decreases steeply (as increased volumes around the particles become non-responsive to RI changes in the medium).

#### 3.2.4. The Effect of the Dielectric Function Used for Gold

**Figure 14:**
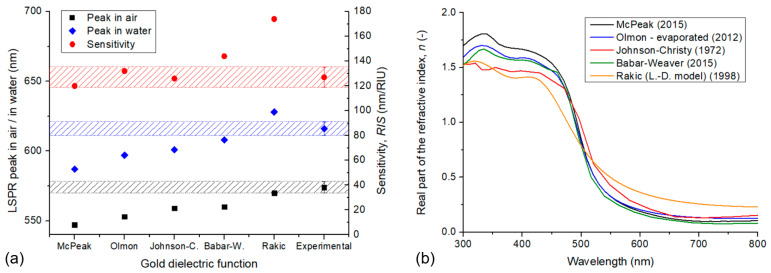
(**a**) LSPR peak positions in air and water, and bulk refractive index sensitivity calculated with different gold dielectric functions for type #2 nanoparticle arrangements (Model no. 7). The dashed areas indicate the range of measured experimental variation for these parameters (data corresponds with Table 4). (**b**) The real part of the complex refractive index in the function of wavelength for the tested datasets.

An important factor, which was not considered up to this point, is the dielectric function used to model the optical response of gold. We selected McPeak’s dataset as it agreed well with experimental data in our previous experience [9]. However, changing the gold dataset affects both the calculated LSPR peak positions and sensitivities significantly. In Figure 14a, five widely used dielectric functions were tested with type #2 particle arrangement (Model no. 7). Besides the previously used McPeak dataset, three experimental sets, namely Olmon’s (evaporated gold, 2012) [20], Johnson and Christy’s (bulk gold, 1972) [21], and Babar–Weaver’s (bulk gold, 2015) [22], we also tested Rakić’s Lorentz–Drude model dataset (1998) [23] as well. The real part of the complex refractive index (*n*) is also plotted in Figure 14b for these datasets, as a reference. 

By comparing the simulation results with the experimental values for this type of nanoparticle arrangement, we can see that although the resulting sensitivity is well in range for the McPeak, Olmon, and Johnson–Christy datasets, the LSPR peak wavelengths are systematically lower, except for the dataset from Rakić. 

Considering the perhaps most widely used Johnson and Christy constants [21], the peak positions in air and water and the sensitivities for the three nanoparticle arrangements result as the following (by using the nominal, average geometrical parameters, as in Table 1 and Model no. 7): type #1: 543 nm, 583 nm and 120 nm/RIU; type #2: 559 nm, 601 nm, and 126 nm/RIU; and type #3 562 nm, 605 nm and 129 nm/RIU, respectively. By comparing the resulting sensitivities with the experimental results in Table 4, the average difference for the three arrangement types is ~2.5 nm/RIU between simulations and the experiment, which is a pretty good match for the sensitivity with this dataset.

#### 3.2.5. Fine-Tuning the Simulations

As discussed previously, if we compare the simulation results with the experimentally obtained LSPR peak position and sensitivity values (see Table 4 and Figure 14a), we can see that although the sensitivities yield a pretty good match, the measured peak positions are significantly lower in the simulated cases. We have seen that the variation in nanoparticle size can account for these small discrepancies in the measured and calculated sensitivities, but not for the significantly different peak positions. As we observed in the previous sections, increasing the nanoparticles’ diameter, decreasing their thickness, or decreasing their interparticle gaps can shift the LSPR peak positions toward longer wavelength. However, as they go hand in hand with the sensitivity, such a modification in the model would also shift the *RIS* out of the experimentally measured range. Table 5 collects and summarizes the effect of the investigated simulation parameters on the calculated LSPR peak positions and sensitivities. Only one investigated geometrical parameter has an opposite effect on the peak positions and sensitivity, namely the particle embedding level. Because of embedding the particles deeper into the substrate, the peak wavelength increases while the sensitivity decreases steeply (as in Figure 13). 

Also, as we demonstrated in Section 3.1.5, the refractive index of the substrate modulates only the peak position without affecting the sensitivities. Initially, the refractive index of the pillars was selected to be 1.45, considering pure SiO_2_ as the base material. However, any impurity or contamination could increase this value, e.g., residual Al_2_O_3_ from the PAA template, stuck between the pillars and the gold. As alumina has a higher refractive index (1.77), its presence inside the near field of the particles (considered as part of the substrate) can increase the LSPR peak position. Also, although all samples were properly cleaned before the experiments, we cannot fully reject the possibility of contaminations on the surface of the gold, which could also increase the measured LSPR peak positions. These effects altogether can be taken into consideration in the simulations as a higher effective substrate refractive index compared to the initial *n*_s_ = 1.45 (pure SiO_2_).

**Figure 15:**
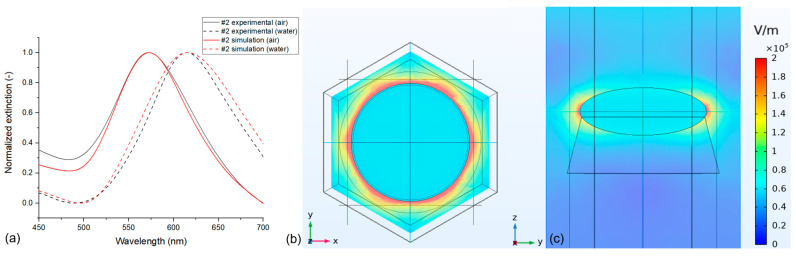
(**a**) Comparison of the experimentally obtained normalized extinction spectra for a specific type #2 nanocomposite sample with the simulated spectra obtained by the optimized parameters. (**b**,**c**) electrical field strength maps in air at the resonance peak of 573 nm.

By knowing the relationship between simulation conditions and LSPR peak positions, sensitivities (Table 5), and the corresponding characteristics (Figure 8, Figure 9, Figure 10, Figure 11, Figure 12, Figure 13 and Figure 14), it is possible to fine-tune the simulations to have an exact match with the experimental results. An example is presented in Figure 15a, where experimentally measured spectra (in air and water) obtained on a type #2 sensor element were used.

For this particular sensor, the measured peak positions were at 573 nm (in air) and 615 nm (in water), corresponding to an *RIS* of 126 nm/RIU. To optimize the simulations, we started with the nominal, experimentally determined geometrical parameters (see Table 1) and selected the Johnson–Christy dataset for gold, as that yields the closest match with the sensitivity based on Figure 14. However, the peak positions were significantly lower with the nominal geometry (559 nm and 601 nm, respectively). Thus, to increase the peak positions, the size of the nanoparticles (namely the diameter/thickness ratio) was increased, in parallel with the embedding rate of the nanoparticles, to compensate for the increased sensitivity. To obtain the best match, as presented in Figure 15a, we used *D*_0_ = 85 nm, *t* = 32 nm, and a *w*/*D*_0_ ratio of 0.95. It is important to emphasize that the used 2 nm deviation from the nominal geometry (for both diameter and thickness) is well within the experimental uncertainty for these parameters, as can be seen in Table 1. Finally, the substrate’s refractive index was set to *n*_s_ = 1.51, which yielded fits within a 1 nm precision for both peaks and sensitivity.

We can consider the complex numerical model, the digital twin of this particular nanocomposite sensor element, that can be used to predict its response in biosensing scenarios, where the thickness and approximate refractive index of the used bioreceptor/target-molecule layers are known [9] and to help in the design and optimization of the nanocomposites for such biosensing applications. 

## 4. Conclusions

The plasmonic behavior of three different hexagonally arranged nanoparticle arrangements consisting of nanoellipsoids was investigated with numerical simulation methods. Finite element and boundary element methods, periodic and nonperiodic boundary conditions with different unit cells were compared. By comparing these models, we found that the BEM and FEM solvers provide well-matching results (within 1 nm precision) for single-particle models and hexagonal particle models with nonperiodic boundary conditions. For periodic boundary conditions, we also showed that the constructed rectangular and hexagonal unit cells yield well-matching results. Based on these optimization results, an FEM model with periodic boundary conditions and hexagonal unit cell was constructed that also takes into account the SiO_2_ pillars under nanoparticle in the realized sensor device. The geometrical parameters of the nanoparticle arrangements, namely the interparticle distance, the particle diameter and thickness, were measured based on SEM and STEM images made on the realized sensors. The effect of these geometrical parameters and also of the used gold dielectric function on the calculated LSPR peak positions and bulk refractive index sensitivities were investigated in detail. We demonstrated that through optimization, the LSPR peak positions and refractive index sensitivities calculated by the models can match the experimentally measured values within a 1 nm (or nm/RIU) precision for the different arrangements. This realized digital twin of the realized nanocomposite plasmonic sensors will be used in the future to optimize the parameters of the fabrication technology in order to maximize sensor performance for the different application areas.

## Figures and Tables

**Table 1:** The average geometrical parameters of the different investigated nanoparticle arrangements; measured based on the SEM and STEM images of Figure 1.

Nanoparticle Arrangement Type	Diameter (*D*_0_, nm)	Thickness (*t*, nm)	Diameter/Thickness (*c*, -)	Interparticle Gap/Particle Diameter (*D*/*D*_0_, -)	Defects per Particle (%)
#1	67.4 ± 2.9	28.8 ± 3.8	2.3 ± 0.4	0.65 ± 0.1	0.9
#2	83.2 ± 3.4	33.6 ± 5.7	2.4 ± 0.4	0.33 ± 0.1	3.2
#3	91.5 ± 4.2	41.7 ± 4.6	2.2 ± 0.4	0.21 ± 0.1	7.0

**Table 2:** Parameters and conditions of the different investigated models. X marks the actual condition in five parameter categories for the seven models.

Model Parameter	Condition	Model Number						
		1	2	3	4	5	6	7
Solver type	FEM	x	x	x	x			x
	BEM					x	x	
Boundary condition (BC)	Nonperiodic	x	x			x	x	
	Periodic			x	x			x
Nanoparticle arrangement type (for nonperiodic BC)	Single particle	x				x		
	Hexagonal (seven particles)		x				x	
Unit cell type (for periodic BC)	Rectangular unit cell			x				
	Hexagonal unit cell				x			x
Substrate	Without substrate	x	x	x	x	x	x	
	With Substrate							x

**Table 3:** Simulation model parameters (solver: FEM/BEM; boundary condition: periodic/nonperiodic; arrangement for nonperiodic simulations: single particle/hexagonal; unit cell for periodic simulations: square unit cell/hexagonal unit cell; substrate: yes/no) and results (LSPR peak position in air and water and bulk refractive index sensitivity) for the three nanoparticle arrangements.

Model Number	Model Details	Nanoparticle Arrangement Type	Peak Air (nm)	Peak Water(nm)	Sensitivity (nm/RIU)
1	FEM nonperiodic single particle	#1	523	569	138
		#2	529	583	162
		#3	528	583	165
2	FEM nonperiodic hexagonal arrangement	#1	530	571	123
		#2	549	595	138
		#3	547	612	195
3	FEM periodic rectangular unit cell	#1	526	566	120
		#2	536	583	141
		#3	540	586	138
4	FEM periodic hexagonal unit cell	#1	526	566	120
		#2	537	584	141
		#3	540	587	141
5	BEM nonperiodic single particle	#1	523	569	138
		#2	529	583	162
		#3	528	583	165
6	BEM nonperiodic hexagonal arrangement	#1	530	571	123
		#2	549	595	138
		#3	546	611	195
7	FEM periodic hexagonal unit cell with substrate	#1	535	569	102
		#2	547	587	120
		#3	551	592	123

**Table 4:** Experimentally obtained LSPR peak positions in air and bulk refractive index sensitivities for the three nanoparticle arrangements.

Nanoparticle Arrangement Type	Number of Samples	LSPR Peak Position in Air (nm)	Average Sensitivity (nm/RIU)
#1	4	579 ± 8	123 ± 14
#2	8	574 ± 4	127 ± 8
#3	8	578 ± 7	124 ± 8

**Table 5:** The effect of the tested simulation parameters on the calculated LSPR peak positions and bulk refractive index sensitivities. **↑** marks direct proportionality, **↓** marks inverse proportionality, while—marks no effect.

Simulation Parameter/Condition	Effect on LSPR Peak Position	Effect on Sensitivity
Nanoparticle diameter (*D*_0_)	↑	↑
Nanoparticle thickness (*t*)	↓	↓
Diameter/thickness ratio (*c* = *D*_0_/*t*)	↑	↑
Interparticle gap (*D*)	↓	↓
Particle gap/diameter ratio (*D*/*D*_0_)	↓	↓
Particle embedding level into the substrate (*w*/*D*_0_)	↑	↓
Substrate refractive index (*n*_*s*_)	↑	-

## Data Availability

The data presented in this study are available on request from the corresponding author.
